# Dry Bean: A Protein-Rich Superfood With Carbohydrate Characteristics That Can Close the Dietary Fiber Gap

**DOI:** 10.3389/fpls.2022.914412

**Published:** 2022-07-26

**Authors:** Mark A. Brick, Adrienne Kleintop, Dimas Echeverria, Sara Kammlade, Leslie A. Brick, Juan M. Osorno, Phillip McClean, Henry J. Thompson

**Affiliations:** ^1^Department of Soil and Crop Sciences, Colorado State University, Fort Collins, CO, United States; ^2^RNA Therapeutics Institute, University of Massachusetts Medical School, Worcester, MA, United States; ^3^Cancer Prevention Laboratory, Colorado State University, Fort Collins, CO, United States; ^4^Department of Plant Sciences, North Dakota State University, Fargo, ND, United States

**Keywords:** soluble dietary fiber, insoluble dietary fiber, oligosaccharides, cultivar variability, dry edible bean

## Abstract

Consumer food choices are often focused on protein intake, but the chosen sources are frequently either animal-based protein that has high fat content or plant-based protein that is low in other nutrients. In either case, these protein sources often lack dietary fiber, which is a nutrient of concern in the 2020–2025 Dietary Guide for Americans. Pulse crops, such as dry edible beans (*Phaseolus vulgaris* L.), are a rich source of dietary protein and contain approximately equal amounts of dietary fiber per 100 kcal edible portion; yet the consumer's attention has not been directed to this important fact. If product labeling were used to draw attention to the similar ratio of dietary protein to dietary fiber in dry bean and other pulses, measures of carbohydrate quality could also be highlighted. Dietary fiber is categorized into three fractions, namely, soluble (SDF), insoluble (IDF), and oligosaccharides (OLIGO), yet nutrient composition databases, as well as food labels, usually report only crude fiber. The objectives of this research were to measure the content of SDF, IDF, and OLIGO in a large genetically diverse panel of bean cultivars and improved germplasm (*n* = 275) and determine the impact of growing environment on the content of DF. Dietary fiber was evaluated using the American Association of Analytical Chemist 2011.25 method on bean seed grown at two locations. Dry bean cultivars differed for all DF components (*P* ≤ 0.05). Insoluble dietary fiber constituted the highest portion of total DF (54.0%), followed by SDF (29.1%) and OLIGO (16.8%). Mean total DF and all components did not differ among genotypes grown in two field environments. These results indicate that value could be added to dry bean by cultivar-specific food labeling for protein and components of dietary fiber.

## Introduction

Pulses, such as dry edible bean (*Phaseolus vulgaris* L.), are known to be an excellent source of dietary protein (Singh, 2017). There is considerable discussion about the current recommended levels of dietary protein given the linkage between protein intake and utilization and age-related muscle loss, frailty, and subsequent aging-related morbidities and mortality (Lonnie et al., 2018). There is also extensive support for and consumer interest in increasing the percent of dietary protein that is obtained from plant sources (Didinger and Thompson, 2020, 2021). Dry bean and other pulses are also rich sources of dietary fiber, which is listed as a dietary factor of concern in the current Dietary Guidelines for Americans (2020–2025) (U.S. Department of Agriculture USDoHaHS, 2020). Inadequate intake of dietary fiber is observed in most economically developed countries. For example, in the United States, ~95% of the population is below the recommended level of dietary fiber intake by >50%, a deficit referred to as the dietary fiber gap (U.S. Department of Agriculture USDoHaHS, 2020). Considerable evidence supports the tenet that closing the dietary fiber gap would reduce morbidity and mortality associated with chronic diseases that currently account for >70% of deaths globally per annum with total amount of dietary fiber being one predictive benefit (Thompson and Brick, 2016; World Health Organization, 2021). Consumption of dry bean and other pulses has been reported to reduce the risk for chronic diseases, presumably in part to their content of dietary fiber (Didinger et al., 2022). It is known that the most health-beneficial form of dietary fiber is that derived from whole foods (reviewed in Thompson and Brick, 2016), which is the form in which most pulses are consumed (Didinger and Thompson, 2020). As reported by us (Thompson and Brick, 2016), the current dietary fiber gap is eliminated in individuals who have a median intake of 277–294 g of pulses per day, an eating behavior that is routine in a subset of individuals in the United States, Canada, and many other countries (Mitchell et al., 2009; Mudryj et al., 2012). What is generally unappreciated is that there is a similar content of protein and dietary fiber in edible dry bean on a dry matter basis or per 100 kcal edible portion (Didinger and Thompson, 2021). Consistent with this fact, in the population data from the United States and Canada that is summarized above (Thompson and Brick, 2016), both protein and dietary fiber intake increased in pulse consumers vs. nonconsumers (Lonnie et al., 2018).

As summarized in Singh (2017), the qualitative and quantitative characteristics of protein in dry edible bean and other pulses have been extensively investigated. In contrast, this is not the case for dietary fiber. A primary reason for the dearth of information is that a consensus definition for dietary fiber was only reached in 2009 and a method implementing that definition in 2011 (CODEX A, 2010; McCleary et al., 2012). Briefly, dietary fiber in food is composed of a complex mixture of carbohydrate polymers that are not hydrolyzed by endogenous enzymes in the small intestine; consequently, they are passed through to the lower digestive tract where they are fermented (Jones, 2014). The American Association of Analytical Chemist (AOAC) categorizes dietary fiber into three components, namely, soluble dietary fiber (SDF), insoluble dietary fiber (IDF), and the indigestible long-chain oligosaccharides (OLIGO) (McCleary et al., 2012). The dietary fiber found in dry edible bean is comprised of all three components (Kleintop et al., 2013). Variation in the content and composition of dietary fiber has been reported among different pulse crops (Chen et al., 2016). However, a comprehensive evaluation of genetic diversity among cultivars of a single pulse crop and the effects of the growing environment on DF content has not been widely conducted. Such work is needed to fully assess the merit of focusing consumer attention on the favorable ratio of protein to fiber in dry bean, as well as to consider how the subfractions of dietary fiber vary among dry bean cultivars. Such work would also set the stage for similar analyses among cultivars of other widely consumed pulses. The objectives of this research were to provide a foundation for further discussion of the issues surrounding the overall importance of high protein pulse crops for the health benefits of the diet. To do this, the content of SDF, IDF, and OLIGO in a large genetically diverse panel of bean cultivars and improved germplasm (*n* = 275) was determined and the impact of growing environment on the content of DF was evaluated.

## Materials and Methods

### Overview

Dietary fiber composition was evaluated on field-grown dry bean seed from a genetically diverse set of bean entries (cultivars/lines) at two field sites. At Fort Collins, CO, a panel of 275 dry bean entries representative of commercial dry bean cultivars/lines currently being grown in North America was evaluated. This panel makes up the “Middle American Diversity Panel” (MDP) used by the Bean CAP research project as a standard genetic population for studies on dry bean (Moghaddam et al., 2018). At Carrington, ND, a subset of the MDP consisting of 57 entries was also evaluated to determine whether DF composition was influenced by environmental effects and estimate Genotype by Environmental Interaction Effects (GXE) for DF and its components. The 57 entries grown in ND were chosen to include all major market classes in the MDP, including pinto, great northern, small white, navy, black, and pink bean cultivars. The seed used in all analyses was harvested from field-grown plots in CO and ND, dried to a constant moisture, and analyzed for dietary fiber content using the AOAC 2011.25 method adopted for dry bean (Kleintop et al., 2013).

### Field Trials

The field trial at the Colorado State University Agricultural Research Development and Extension Center in Fort Collins, CO (40.58◦ N, 105.08◦ W), was used to evaluate the entire MDP. The elevation at this site is 1,554 masl and has an average of 142 frost-free days per year. The soil at the site is a Fort Collins loam (superactive, mesic Aridic Haplustalfs). The experimental design was a randomized-complete-block design with two replicates, and entries randomized in blocks. Experimental units were two-row plots, 6.4 m in length, spaced 0.76 m apart. The previous year's crop on this field was corn (*Zea mays* L.). Urea (46-0-0) was broadcast pre-plant at a rate of 50 kg N ha^−1^ and incorporated with a roller harrow. Herbicides labeled for dry beans were used to control weeds throughout the growing season. Planting occurred on 4 June 2011, using a precision air-planter at a seeding rate of ~195,000 seeds ha^−1^. The trial was furrow irrigated throughout the growing season and received 506 mm of rainfall plus irrigation during the growing season. At harvest maturity, two 1-m sections of each experimental unit were hand-pulled 87–99 days after planting depending on specific maturity of the entry. Plant maturity was determined by visually estimating the time when 50% of the pods had discolored from green to light tan/brown. All plots were threshed with a Vogel stationary thresher and weighted.

Field plots located at the North Dakota State University Carrington Research and Extension Center (47.4◦ N, 99.1◦ W) were used to evaluate a subset of 57 entries from the MDP to determine whether location influenced the content of DF and its components. The elevation at this site is 484 masl and has an average of 121 frost-free days. No fertilizer was added to the plots because pre-plant soil analyses determined that soil residual N content was 30 kg ha^−1^, an amount considered adequate for commercial bean production in ND. Field plots were maintained with limited irrigation, and the total applied growing season water was 395 mm (rainfall + irrigation). Soil at the Carrington site is a complex Letcher fine sandy loam and Hecla fine sandy loam. The previous year's crop at the site was spring wheat (*Triticum aestivum* L.). The experiment was an alpha-design with two replicates for each entry. Experimental units were two-row plots, 6.4 m in length spaced 0.76 m apart. Labeled herbicides were used to control weeds throughout the growing season. Planting occurred on 19 May 2011, using a four-row John Deere precision planter at a seeding rate of ~195,000 seeds ha^−1^. At harvest maturity, plants were removed from the soil with a custom Pickett^®^ rod-cutter, windrowed, and threshed ~5 days later with an experimental plot thresher (Almaco^®^ SPC 20). Since harvest maturities among entries differed, seed harvest among entries was made progressively between 5 and 20 September.

### Preparation of Seed Samples for Fiber Analysis

Seed samples from each replicate of each dry bean entry from the two locations were dried for 24 h in a convection oven at 40◦C to bring all seeds to ~6% moisture content. After drying, the seed samples were weighed and ~1 g of dry bean seed was transferred to 50-ml conical tubes. The seed samples were soaked in tubes containing 8 ml of distilled water for 14 h. After soaking, the tubes were then transferred to an autoclave and cooked for 65 min. The cooked samples were then homogenized in the tube and frozen at −80◦C until analysis.

### Fiber Analysis

The Integrated Total Dietary Fiber Assay AOAC Method 2011.25, as modified by Kleintop et al. (2013), was used for analysis of dietary fiber components (McCleary, 2007). An abbreviated summary of the procedure is as follows: frozen homogenized seed samples were thawed at room temperature and transferred to a 100-ml glass bottle. A buffer solution and α-amylase were added to each glass bottle with 20 ml of water and placed into water bath and incubated for 16 h. After 16 h, the glass bottles were removed from the water bath and the pH was adjusted to ~8.0. After the samples were allowed to cool, a protease solution was added to each bottle. The bottles were placed in a shaking incubation water bath for 30 min. Following incubation with protease, the pH of each sample was adjusted to ~4.3. The contents of the glass bottle were vacuum-filtered in fritted glass crucibles that contained ~1 g of Celite^®^. After filtration, the precipitate and Celite^®^ residues were collected and dried. Residues were weighed and the amount or ash and protein determined to obtain only IDF values. The filtrate from the IDF filtration was mixed with a solution of ETOH to allow the SDF to precipitate for 60 min at room temperature. The contents were poured into a pre-wetted fritted crucible containing Celite^®^ under vacuum suction as described in the first gravimetric filtration. The SDF was retained on the crucible as filtrate and the oligosaccharides in the filtrate passed through the filter and collected into a 1-L flask. The filtrate volume was recorded, and 45 ml of the filtrate was saved and stored at −80◦C for later analysis of oligosaccharide content. The crucibles containing the SDF residue were dried in a convection oven at 105◦C overnight. The crucibles were removed and placed in a desiccator for 1 h, then weighed to the nearest 0.1 mg. To determine the mass of the residue, the mass of crucible and Celite^®^ was subtracted from this value. Residues were saved for ash and protein corrections as with the first filtration. The percentage of IDF or SDF was calculated using the residue weights from the two filtrations and subtracting the amount of protein and ash in the residue. The filtrate retained after the second gravimetric filtration contained the oligosaccharides that were analyzed by high-performance anion-exchange chromatography.

### Statistical Analysis

An analysis of variance (ANOVA) was carried out to compare the main effects of entry (cultivar/line) and location for all variables using the Proc GLM procedure in SAS version 9.2 (SAS Institute Inc., Cary, NC, USA). Two replicates per entry were used for all variables. Replicates were considered a random-factor and locations and entries as fixed-factor models. In the ANOVA, the *F*-test was used to test the main effects of entry, location, and interaction effects. The least significant difference mean comparison method (*P* ≤ 0.05) was used to determine significance among entry and location means for all variables.

## Results

### Total Dietary Fiber Content of Dry Beans Entries

Dry bean entries (275) grown at Fort Collins differed (*P* ≤ 0.05) for all DF variables, including IDF, SDF, OLIGO, and TDF (Tables 1, 2). Among the DF components, IDF constituted the highest portion (54.0%), followed by SDF (29.1%) and OLIGO (16.8%). Total dietary fiber varied from 22.82 to 30.23% among entries (Table 1). A black-seeded cultivar, PR0443-151, had the highest TDF content with 30.23%, and a pinto-seeded entry, AC Pintoba, had the lowest with 22.82%. These results are similar to those reported by Kleintop et al. (2013), where they found that TDF ranged from 20.0 to 27.0% in a set of 30 dry bean cultivars. The higher levels and range of TDF found in this study are likely due to the evaluation of a more genetically diverse set of entries.

**Table 1:** Range, grand means, and the five entries ranked highest and lowest for total dietary fiber (TDF) content and their respective components, insoluble dietary fiber (IDF), soluble dietary fiber (SDF) and total oligosaccharide (T OLIGO) content among entries (275) grown in Colorado.

	**IDF**	**SDF**	**T OLIGO**	**TDF**
	**% Dry weight of seed**			
Range	11.30–18.88	3.98–10.42	3.50–5.98	22.82–30.23
Grand mean	14.14	7.64	4.40	26.19
**Entry**	**Entries ranked with highest TDF**			
PR0443-151	16.91	8.19	5.13	30.23
IP08-2	15.73	9.48	4.85	30.06
ND021717	16.23	7.57	5.98	29.78
AC Resolute	15.88	8.33	5.24	29.45
Max	16.12	8.00	4.46	28.59
	**Entries ranked with lowest TDF**			
T9905	12.59	7.46	3.53	23.59
Norstar	12.73	6.44	4.26	23.43
BelMiNeb-RMR-7	11.77	7.09	4.49	23.36
Topaz	12.45	6.70	4.03	23.18
AC Pintoba	11.44	7.02	4.36	22.82
LSD_(0.05)_	0.12	0.10	0.05	0.32

**Table 2:** Range, grand means, and five dry bean entries with the highest and lowest respective raffinose, stachyose, verbascose, and total oligosaccharide (OLIGO) content among entries (275) grown in Colorado.

	**Raffinose**	**Stachyose**	**Verbascose**	**Total OLIGO**
	**% Dry weight of seed**			
Range	0.27–0.92	2.99–5.16	0.02–0.23	3.50–5.98
Grand mean	0.45	3.85	0.10	4.40
**Five entries with the highest respective oligosaccharide content**				
	0.92	5.16	0.23	5.98
	0.85	4.90	0.23	5.45
	0.85	4.78	0.20	5.39
	0.79	4.70	0.20	5.32
	0.79	4.69	0.20	5.26
**Five entries with the lowest respective oligosaccharide content**				
	0.30	3.10	0.04	3.60
	0.30	3.07	0.04	3.57
	0.30	3.03	0.04	3.55
	0.28	3.00	0.03	3.53
	0.27	2.99	0.02	3.50
LSD _(0.05)_	0.05	0.05	0.01	0.05

### Insoluble and Soluble Dietary Fiber Content

Insoluble dietary fiber content among the 275 entries ranged from 11.30 to 18.88%, with a mean of 14.14% (Table 1). Soluble dietary fiber content ranged from 3.98 to 10.42% with a mean of 7.64%. The level of differences observed among the highest and lowest entries for both IDF and SDF also suggests that adequate genetic variation exists for improvement of these traits in a breeding program. In fact, for SDF the magnitude of difference from low to high is >100%.

### Raffinose, Stachyose, Verbascose, and Total Oligosaccharide Content

Total oligosaccharide content among entries ranged from 3.50 to 5.98% dry weight (Table 2). Among the oligosaccharide components, stachyose constituted the highest portion (87.50%), followed by raffinose (10.2%) and verbascose (0.02%). Raffinose, stachyose, and verbascose content ranged from 0.27 to 0.92% with mean of 0.45%, 2.99 to 5.16% with mean of 3.85%, and 0.02 to 0.23% with mean of 0.10%, respectively. A black seeded entry (ND021717) had the highest total oligosaccharide content with 5.98%, and a small red seeded entry (F07-449-9-3) had the lowest content with 3.50%. These results indicate that genetic variability exists in the MDP for these DF components.

### Environmental Effects on DF and Interactions With Entries

The food production industry has a vested interest in producing food crops with uniformly high nutritional value. One issue facing the food industry is that environmental conditions that differ during the growing season or across location can alter the content of these nutrients. For some nutrients, the nutrient level may vary for a given cultivar across environments and for others the nutrient content of a cultivar may remain stable over environments. These effects are known as “Genotype by Environmental Interactions.” It is desirable to use cultivars that have a high nutrient content and do not change performance (interact) across environments. The occurrence of interactions between cultivars and environments can be tested by growing several cultivars at locations, then conducting an *F*-test for the interaction in the ANOVA. If the performance of cultivars is unstable across environments, the *F*-test for the interaction will be significant at a chosen error rate, and if the cultivars are stable the *F*-test will not be significant. For this reason, we tested cultivars grown in Fort Collins, CO, and Carrington, ND, to determine whether DF components for cultivars performed the same across environments or interacted with the environment.

#### Insoluble, Soluble, and Total Dietary Fiber Means and Interactions

**Figure 1:**
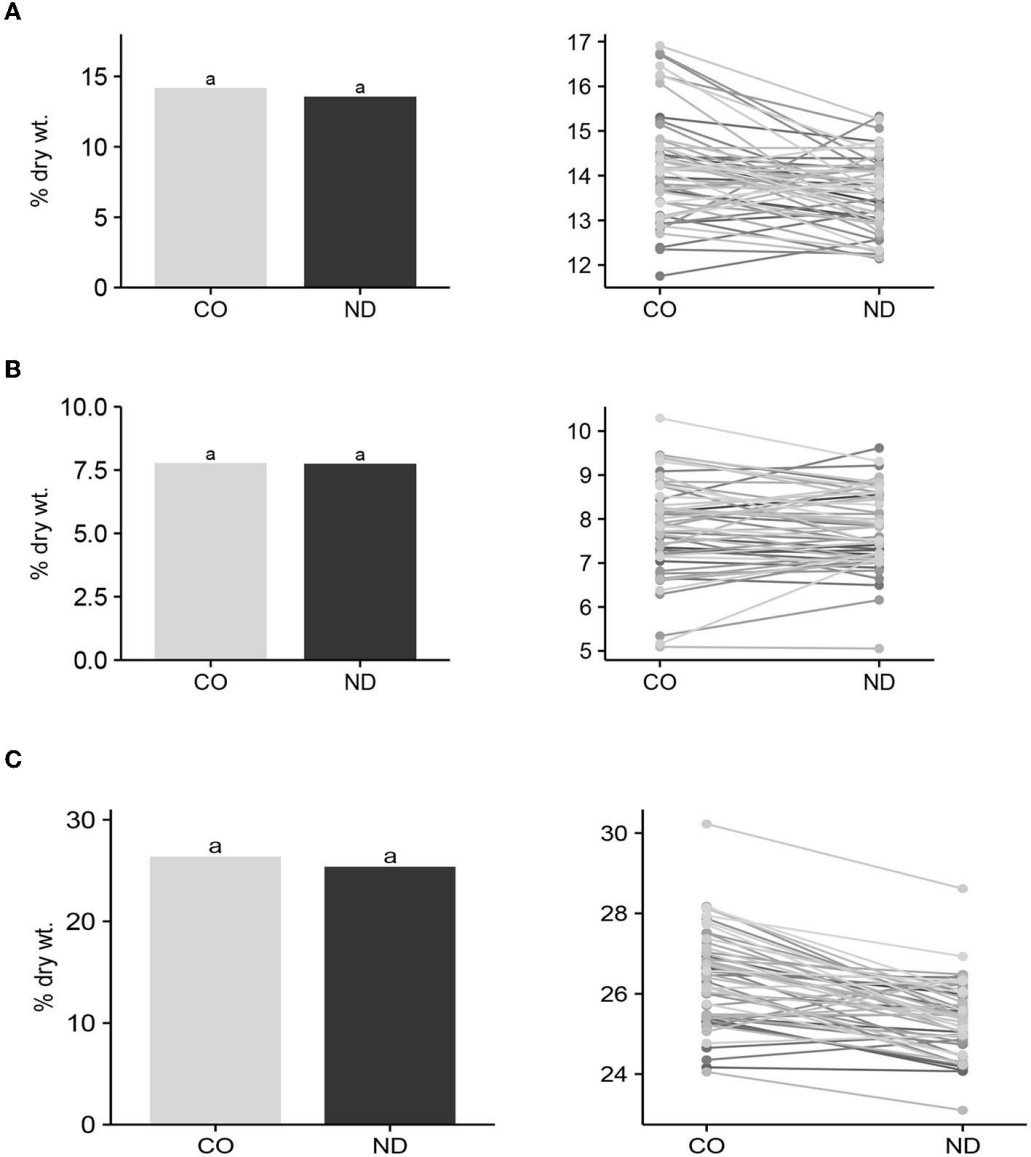
Mean insoluble **(A)**, soluble **(B)** and total dietary fiber **(C)** content among cultivars/lines (*n* = 57) grown in Colorado (CO) and North Dakota (ND), and graphic response of content among entries over locations. Bar means with the same lowercase letter are not significantly (*P* ≤ 0.05) different between locations.

Mean IDF, SDF, and TDF among entries (*n* = 57) between seed produced in CO and ND did not differ (*P* > 0.05) (Figure 1). Furthermore, the *F*-test for the interactions of entries with locations was not significant (*P* > 0.05) for any of the three DF variables. These results suggest that DF production and its components are relatively stable for cultivars across environments. Hence, entries with high IDF, SDF, or TDF at one location should also have high levels of DF at another location. Since this test was conducted on only two locations, it would be good to further validate this conclusion at additional locations.

#### Raffinose, Stachyose, Verbascose, and Total Oligosaccharide Means and Interactions

**Figure 2:**
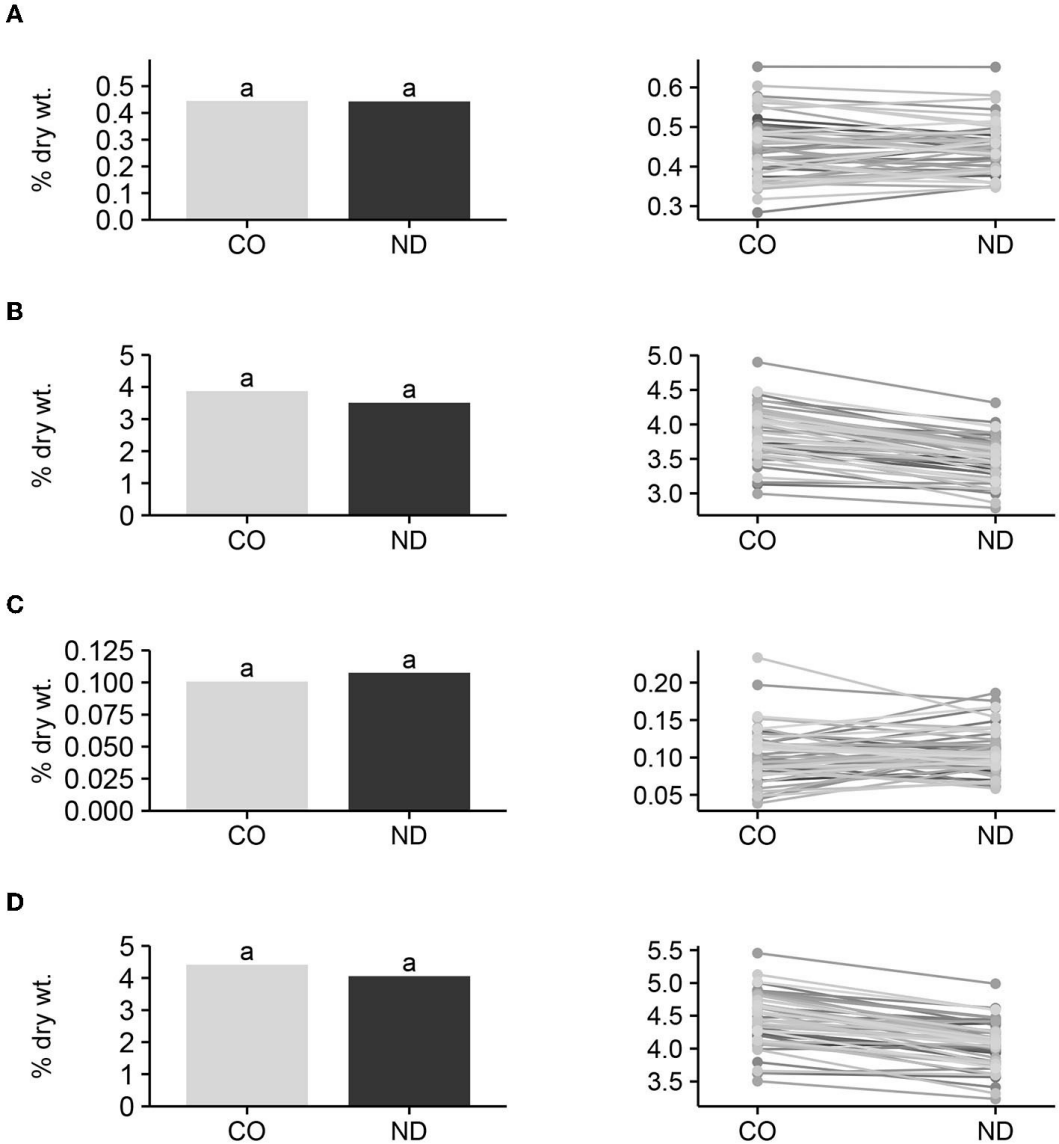
Mean raffinose **(A)**, stachyose **(B)**, verbascose **(C)**, and total oligosaccharide **(D)** content among entries (*n* = 57) grown in Colorado (CO) and North Dakota (ND), and graphic response of content among entries over locations. Bars with the same lowercase letter are not significantly (*P* ≤ 0.05) different between locations.

Mean raffinose, stachyose, verbascose, and total oligosaccharide content among entries did not differ (*P* > 0.05) between seed produced in CO and ND (Figure 2), suggesting that location effect is not an important factor that contributes to variation in DF content. Furthermore, the *F*-test for the interactions of entries and locations was not significant (*P* > 0.05) for any of the four variables, and the average performance of individual entries did not change over locations. Therefore, in general, entries with high IDF, SDF, or TDF at one location also had high levels of DF at the alternative location. Since this research was conducted on only two locations, it would be good to further validate this conclusion in additional locations. These results are encouraging in that the environment did not influence the entry performance of IDF, SDF, or TDF, and dry bean producers should have some confidence that selection for high IDF, SDF, or TDF cultivars based on performance in one location would also have high respective DF in an alternative environment. This would also allow the dry bean industry to increase TDF in bean products by selecting for and growing high TDF cultivars for commercial bean production across a range of environments.

## Discussion

Edible dry bean is a global crop that is organized into market classes that have specific characteristics by which they are identified and marketed to consumers. In the United States, 10 market classes predominant. Inspection of nutrient labeling information for these market classes reveals differences in the amount of protein and fiber per serving among market classes, but in all cases their ratio approaches 1:1 in any given market class. However, within each market class there are generally thousands of cultivars, although those grown commercially are limited in number mostly based on favorable agronomic traits. The way in which the food system operates, i.e., at the level of market class, hides the variation that exists among cultivars in the content of all bioactive components, including protein and fiber. Herein, that variation is unmasked for components of dietary fiber.

All the bean cultivars evaluated in this study were observed to be excellent sources of dietary fiber; however, the fact that there was a 28% difference between the amount of TDF between cultivars with the lowest and highest TDF indicates an unappreciated opportunity for both consumers and producers. For example, if an individual were to follow the dry bean-rich diet investigated in the polyp prevention trial (Lanza et al., 2006), they would consume at least 30 g of TDF per day from the highest cultivars. In addition, producers could add value to the bean varieties they market by noting a high fiber cultivar and the feasibility of doing this is supported by the lack of a significant effect due to the environment in which the cultivar was grown. While confirmation is needed, it is likely that some cultivars would have high fiber as well as high protein content. This creates the opportunity for value-added marketing for protein, fiber, and their ratio. This would likely be attractive to individuals of all ages but for diverse reasons, including cardiovascular fitness, gut health, chronic disease risk mitigation, and environmental sustainability (Didinger and Thompson, 2020, 2021; Didinger et al., 2022).

The study reported herein expands the consideration of total dietary fiber to include the analysis of fiber subfractions. This is done by recognizing that there is extremely limited information available with regard to the agronomic or the biomedical activity of the individual components of total dietary fiber. In this regard, the article is a call to action. Clearly, there is sufficient variation at the level of cultivar to identify cultivars with specific fiber subfraction characteristics, although the argument can be made that more information is needed to guide such choices. Emergent areas include how cooking time is impacted by various fiber fractions, whether protein digestibility or content is related to specific fiber fractions, and relative importance of the prebiotic activity of each fraction for gut health (Mongeau et al., 1989; Lutsiv et al., 2021; Sadohara et al., 2022). With such guidance, the opportunity would exist for plant breeders to select cultivars for either IDF or SDF in a breeding program. However, this would likely reduce the component not under selection pressure because IDF and SDF content have been reported to be negatively correlated (*r* = −0.32, *P* < 0.001) (Moghaddam et al., 2018). Nonetheless, based on a genome-wide association study to identify potential candidate genes that control IDF and SDF, no common candidate genes were found that contributed to both SDF and IDF (Moghaddam et al., 2018). In that study, most of the candidate genes associated with IDF content were related to cellulose synthesis and microfibril organization; cell wall synthesis, degradation, and remodeling; and hemicellulose remodeling. The candidate genes associated with SDF were related to soluble polysaccharide synthesis. It is unclear how IDF or SDF (or its proportion) would change if selection were imposed solely on TDF. Relative to the content of OLIGO in dry bean, there has been interest in reducing its concentration because of concerns about flatulence. Bean OLIGOs are primarily galactans, and humans lack alpha-galactosidase to digest them. Consequently, oligosaccharides are fermented by bacteria in the human digestive tract, and the gas produced causes flatulence, and sometimes digestive discomfort (Thompson, 2019). However, more details about the prebiotic effects of oligo-galactans are needed before the value of a breeding program to reduce their content can be assessed.

## Conclusion

Given the importance of value-added traits for product marketing, the variation found in DF and its components in this study indicates that plant breeders or commercial grain handlers should be able to alter the content of DF in dry bean products either by breeding for improved TDF cultivars or by selecting cultivars that currently have a higher DF. Since the highest TDF cultivar observed in this study contained ~28% more TDF than the lowest cultivar, it is plausible that cultivars currently on the market could contribute to a substantial increase in DF in dry edible bean products if they were tested for their stability of performance across locations. These results support the opportunity to use source-identified bean cultivars to increase DF in bean products. If cultivar-specific DF labeling is adopted and is associated with a value-added price premium, the dry bean industry would be incentivized, *via* breeding and selection, to increase DF in bean products with the added benefit of having high protein content.

## Data Availability Statement

The original contributions presented in the study are included in the article/supplementary material, further inquiries can be directed to the corresponding author/s.

## Author Contributions

MB and HT: conceptualization and writing—original draft. MB, DE, JO, and HT: data curation. MB, SK, and HT: formal analysis. MB, JO, PM, and HT: funding acquisition, project administration, and resources. MB, AK, JO, and HT: investigation. MB, DE, LB, and HT: methodology. MB, JO, and HT: supervision. MB, SK, and LB: validation. SK: visualization. MB, AK, DE, SK, LB, JO, and HT: writing—review and editing. All authors contributed to the article and approved the submitted version.

## Funding

This study was supported by the USDA, National Institute of Food and Agricultural (NIFA), Agriculture and Food Research Initiative (AFRI), Project #2009-01929, and the Colorado State University and North Dakota State University Agricultural Experiment Stations.

## Conflict of Interest

The authors declare that the research was conducted in the absence of any commercial or financial relationships that could be construed as a potential conflict of interest.

## Publisher's Note

All claims expressed in this article are solely those of the authors and do not necessarily represent those of their affiliated organizations, or those of the publisher, the editors and the reviewers. Any product that may be evaluated in this article, or claim that may be made by its manufacturer, is not guaranteed or endorsed by the publisher.
